# Tumor Microenvironment in Mixed Neuroendocrine Non-Neuroendocrine Neoplasms: Interaction between Tumors and Immune Cells, and Potential Effects of Neuroendocrine Differentiation on the Tumor Microenvironment

**DOI:** 10.3390/cancers14092152

**Published:** 2022-04-26

**Authors:** Junichi Tsunokake, Fumiyoshi Fujishima, Hirofumi Watanabe, Ikuro Sato, Koh Miura, Kazuhiro Sakamoto, Hiroyoshi Suzuki, Takashi Sawai, Yuko Itakura, Tatsuya Hoshi, Atsushi Kunimitsu, Takuro Yamauchi, Ryujiro Akaishi, Yohei Ozawa, Toshiaki Fukutomi, Hiroshi Okamoto, Chiaki Sato, Yusuke Taniyama, Takashi Kamei, Hironobu Sasano

**Affiliations:** 1 Department of Surgery, Tohoku University Graduate School of Medicine, 1-1 Seiryo-machi, Aoba-ku, Sendai 980-8574, Japan; atsushi.kunimitsu@surg.med.tohoku.ac.jp (A.K.); taku.y.xc5@surg.med.tohoku.ac.jp (T.Y.); ryujiro.a@surg.med.tohoku.ac.jp (R.A.); yohei.ozawa@surg.med.tohoku.ac.jp (Y.O.); t-fukutomi@med.tohoku.ac.jp (T.F.); hi-ok@surg.med.tohoku.ac.jp (H.O.); schiaki@surg.med.tohoku.ac.jp (C.S.); yusuketaniyama@med.tohoku.ac.jp (Y.T.); tkamei@surg.med.tohoku.ac.jp (T.K.); 2 Department of Pathology, Tohoku University Hospital, 1-1 Seiryo-machi, Aoba-ku, Sendai 980-8574, Japan; ffujishima@patholo2.med.tohoku.ac.jp (F.F.); hi.wa@patholo2.med.tohoku.ac.jp (H.W.); hsasano@patholo2.med.tohoku.ac.jp (H.S.); 3 Department of Pathology, Miyagi Cancer Center, Natori 981-1293, Japan; sato-ik510@miyagi-pho.jp; 4 Department of Surgery, Miyagi Cancer Center, Natori 981-1293, Japan; kou-miura@miyagi-pho.jp; 5 Department of Pathology, Osaki Citizen Hospital, Osaki 989-6183, Japan; ksakamoto-och@h-osaki.jp; 6 Department of Pathology, Sendai Medical Center, Sendai 983-8520, Japan; suzuki.hiroyoshi.vc@mail.hosp.go.jp; 7 Department of Pathology, Sendai Open Hospital, Sendai 983-0824, Japan; sawai@wonder.ocn.ne.jp; 8 Department of Pathology, Japanese Red Cross Ishinomaki Hospital, Ishinomaki 986-8522, Japan; itak@patholo2.med.tohoku.ac.jp; 9 Department of Internal Medicine, Kesennuma Citizen Hospital, Kesennuma 988-0181, Japan; t-hoshi@kesennuma-hospital.jp

**Keywords:** mixed neuroendocrine non-neuroendocrine neoplasms, tumor microenvironment, tumor infiltrating lymphocyte, neuroendocrine differentiation, angiogenesis, immune suppression

## Abstract

**Simple Summary:**

The neuroendocrine differentiation of tumors is considered to influence the tumor microenvironment through the secretion of various hormones or growth factors. However, cases of neuroendocrine and non-neuroendocrine neoplasms are difficult to compare because of the potential differences in systemic and local immune environments. The analysis of mixed neuroendocrine non-neuroendocrine neoplasms, in which neuroendocrine and non-neuroendocrine components are present in the same tumor, could provide important insights into the effects of neuroendocrine differentiation on tumor microenvironments. However, to the best of our knowledge, this has not been reported yet. Here, we compared the status of the tumor tissue microenvironment, including the infiltrating lymphocytes present, in the neuroendocrine and non-neuroendocrine areas of the same tumor. Factors related to neoangiogenesis and the suppression of tumor immune reactions were more abundant in neuroendocrine than in non-neuroendocrine areas. Therefore, neuroendocrine and non-neuroendocrine tumors differ with respect to the characteristics of both tumor cells and the tumor microenvironment.

**Abstract:**

The tumor microenvironment is considered to play a pivotal role in various human malignancies. Neuroendocrine and non-neuroendocrine neoplasms are considered to have different tumor microenvironments. However, owing to differences in the systemic and/or local immune statuses, tumor microenvironments in different patients may be difficult to compare. Mixed neuroendocrine non-neuroendocrine neoplasms (MiNENs), although rare, could be useful for exploring the effects of neuroendocrine differentiation on the tumor microenvironment, because both neuroendocrine and non-neuroendocrine components are present in the same tumor. Here, we examined 33 cases of histologically confirmed MiNENs and evaluated the influence of neuroendocrine differentiation on the tumor microenvironment by comparing tumor-infiltrating lymphocytes, tumor-associated macrophages, and other relevant factors in the two components the same tumor. The immunoreactivity of those examined above was evaluated quantitatively. The values of vasohibin-1-positive density (*p* < 0.0001) and immunoreactivity (*p* < 0.0001) (representing the neoangiogenesis status) were significantly higher in neuroendocrine as compared to non-neuroendocrine areas of the same tumors. In addition, the Foxp3/CD8 (*p* = 0.0717) and the PD-1/CD8 ratios (*p* = 0.0176) (representing tumor immunity suppression) tend to increase in neuroendocrine carcinomas. Immunoreactivity of CD163, a marker of M2-like macrophages, was also higher in the neuroendocrine areas. Our findings indicate that neuroendocrine and non-neuroendocrine tumors differ from each other with respect to the characteristics of both tumor cells and the tumor microenvironment.

## 1. Introduction

Neuroendocrine neoplasm (NEN) is a malignant neoplasm derived from neuroendocrine cells distributed in different organs [1,2]. NENs are currently classified as well-differentiated neuroendocrine tumors (NETs) and poorly differentiated neuroendocrine carcinomas (NECs) [3]. NETs and NECs are further classified as G1/G2/G3 and small/large cell carcinomas, respectively, based on their mitotic rate (mitoses/mm^2^) or Ki-67 index (%) in the former and morphological features in the latter [3,4]. Composite tumors with neuroendocrine and non-neuroendocrine differentiation, including adenocarcinomas or squamous cell carcinomas, are extremely rare. The World Health Organization (WHO) initially defined these tumors as mixed adenoneuroendocrine carcinoma, a composite tumor comprising NEN and adenocarcinoma components [5,6,7,8]. These composite tumors were subsequently termed mixed neuroendocrine non-neuroendocrine neoplasms (MiNENs) in the WHO 2019 classification, based on the presence of at least 30% of either component in the same tumor [9]. MiNENs have also been reported to show clinical prognosis or outcomes similar to pure NEC, but this could be different among the organs in which they occur [10,11,12,13]. In addition, despite complete resection, MiNENs often recur after surgery and frequently develop therapeutic resistance to post-operative chemotherapy, owing to the different responses of the neuroendocrine and non-neuroendocrine components to chemotherapeutic regimens [9]. Various factors and components of the tumor microenvironment, including neoangiogenesis and the status of tumor-infiltrating lymphocytes (TILs) and tumor-associated macrophages (TAMs) related to tumor immunity, have been reported to play important roles in the clinical and/or biological behavior of cancer cases [14,15,16,17,18,19,20]. For example, the increased infiltration of CD8-positive lymphocytes into tumor tissues was reported to be associated with better clinical outcome of the patients, whereas that of CD4- and Foxp3-positive lymphocytes with their worse prognosis [21,22,23,24,25,26,27]. da Silva et al. also reported no significant differences in the intra-tumoral CD3- and CD8-positive lymphocyte infiltration status between gastrointestinal and pancreatic NETs [25]. However, the status of infiltrating lymphocytes and macrophages has not been well studied in gastrointestinal or pancreatic NECs. The tumor microenvironment has been considered to be influenced not only by the characteristics of carcinoma cells but also by the cross-talk between carcinoma and stromal cells, although various systemic factors could influence the tumor microenvironment. Therefore, it is generally difficult to compare the tumor microenvironments among different cases of NETs and non-NETs. In this regard, MiNENs could be an ideal model for exploring the influence of neuroendocrine differentiation on the tumor microenvironment, because both components are concurrently present in the same tumor of the same patient. In this study, we immunolocalized the factors associated with the tumor microenvironment in MiNENs and evaluated the influence of neuroendocrine differentiation on TILs, TAMs, and other molecules present in the tumor microenvironment by separately examining the tumor microenvironment of the two different areas above. Results firstly revealed that the neuroendocrine and non-neuroendocrine components of MiNENs were substantially different in the status of the factors associated with their corresponding tumor microenvironment.

## 2. Materials and Methods

### 2.1. Tissues and Patient Characteristics

In our present study, we evaluated 33 cases of gastroenteropancreatic (GEP)-MiNEN, where the patients underwent surgical resection between January 2001 and June 2021, after careful histopathological confirmation including immunohistochemistry. All non-neuroendocrine components in those cases were either adenocarcinomas or mucinous carcinoma. We excluded the cases where patients had received neo-adjuvant chemotherapy prior to surgery or where squamous cell carcinoma was the non-NEC component. These cases were retrieved from the surgical pathology files of Tohoku University Hospital, Sendai, Japan; Miyagi Cancer Center, Natori, Japan; Osaki Municipal Hospital, Osaki, Japan; Sendai Medical Center, Sendai, Japan; Sendai Open Hospital, Sendai, Japan; Japanese Red Cross Ishinomaki Hospital, Ishinomaki, Japan; and Kesennuma Citizen Hospital, Kesennuma, Japan. Tissues fixed in 10% formalin and embedded in paraffin blocks were retrieved and carefully re-examined, with emphasis on the adherence to the stringent criteria for MiNENs. Based on their definition, all neuroendocrine or NEN components were NECs. The non-NEC components examined included adenocarcinomas, squamous cell carcinomas, and mucinous carcinomas. The NEC components of the cases were carefully identified based on the immunolocalization of neuroendocrine markers (at least one positive among synaptophysin, chromogranin A, or insulinoma-associated 1). These neuroendocrine markers were also reported to be abundant in some NECs, which was consistent with the results of our present study [28]. The histologically identified transition between tumor cells with neuroendocrine and non-neuroendocrine differentiation was carefully evaluated in all 33 cases. The details of the neuroendocrine and non-neuroendocrine components examined in this study are summarized in Table 1. The research protocol of the study was approved by the ethical committee of Tohoku University School of Medicine (accession number 2020-1-889) and other institutions.

### 2.2. Immunohistochemistry

One representative tissue section containing adequate NEN and non-NEN components was selected in all the cases examined after careful histological evaluation and the corresponding serial tissue sections (thickness: 3–4 μm) were prepared. The slides were deparaffinized with xylene and dehydrated using graded ethanol solutions. The immunohistochemistry protocols used in this study are summarized in Table 2.

### 2.3. Evaluation of Immunoreactivity

**Figure 1:**
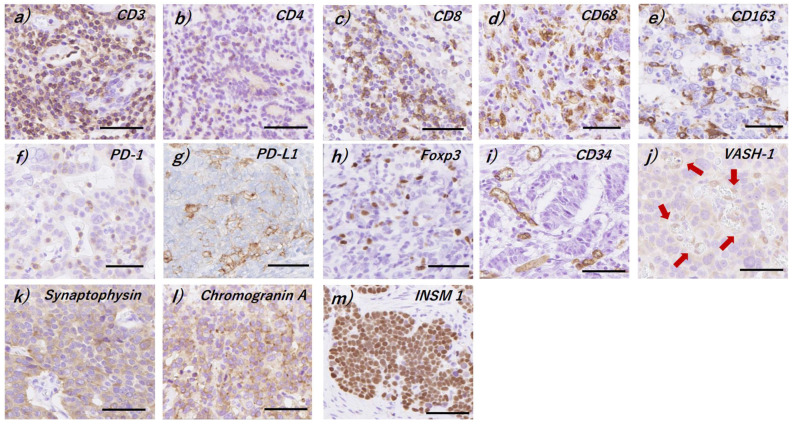
Representative illustration of immunohistochemical staining. (**a**) CD3, (**b**) CD4, (**c**) CD8, (**d**) CD68, (**e**) CD163, (**f**) PD-1, (**g**) PD-L1, (**h**) Foxp3, (**i**) CD34, (**j**) vasohibin-1 (VASH-1), (**k**) synaptophysin, (**l**) chromogranin A, and (**m**) insulinoma-associated 1 (INSM1). Arrows: VASH-1-positive micro-vessels. Bars: 50 μm.

**Figure 2:**
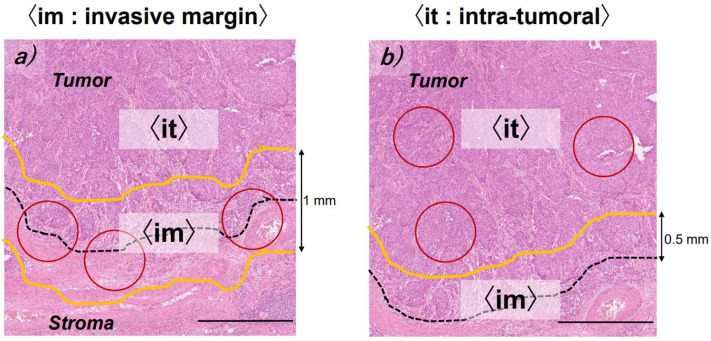
Representative examples of the evaluation of lymphocytes or macrophages testing positive for different markers. (**a**) Invasive margin area (the area centered on the border separating the tumor and normal stroma with an extent 1 mm), (**b**) intra-tumoral area (from the tumoral side to the invasive margin on the tumoral side) →The average value from three areas (circle of 0.75 mm^2^) was calculated in each case. Bars: 1 mm.

**Figure 3:**
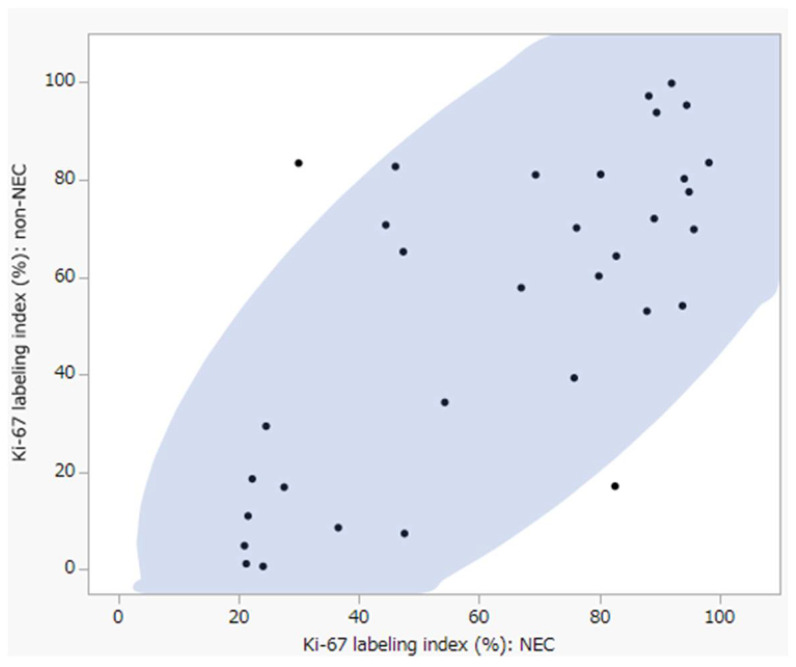
The plot of Ki-67 labeling index each of non-NEC and NEC component (vertical: non-NEC, horizontal: NEC). Non-NEC: 1–99%, NEC: 21–98%.

All the slides with immunohistochemical staining were digitally scanned using the Nanozoomer S360 (C13220-01, Hamamatsu Photonics, Shizuoka, Japan) for digital image analyses (DIA). DIA was subsequently performed using the HALO^®^ Membrane v1.7 (Indica Laboratories, Corrales, NM, USA). In this study, we excluded the areas containing necrosis and lymph follicles as well as those containing less than 100 cells in the field. The representative immunoreactivity obtained is illustrated in Figure 1a–m. The CD3, CD4, CD8, CD68, CD163, PD-1, and Foxp3 immunoreactivities were tentatively examined in three different areas (×200: 0.75 mm^2^ circle), with the highest positive cell counts observed in both invasive margins and intra-tumoral areas, following which the average in all the cases examined was calculated (Figure 2) [29]. We applied nuclear segmentation and quantification algorithms to evaluate CD3, CD4, CD8, and PD-1 immunoreactivity [30,31]. Cytoplasm segmentation quantification algorithms were used to evaluate the CD68 and CD163 immunoreactivity [32]. Nuclear segmentation quantification algorithms were also applied to evaluate Foxp3 immunoreactivity [33]. PD-L1 immunoreactivity was interpreted as positive if immunoreactivity was observed in the membrane in more than 1% of the total tumor area. CD34 immunoreactivity was evaluated in terms of micro-vessel density (MVD) in five different tumor areas (×200: 0.75 mm^2^ circle) [34]; the number of the vessels with vascular endothelial cells positive for CD34 was determined, and the area with the highest MVD was tentatively selected [34]. Vasohibin-1 (VASH-1) immunoreactivity was determined by counting the number of the vessels with vascular endothelial cells expressing VASH-1 in the MVD area (VASH-1 density), and the ratio of VASH-1 density to MVD (VASH-1 expression: VASH-1/MVD) was calculated [34]. Only the CD34- and VASH-1-positive vasculature was quantified using a combination of DIA and manual methods in this study. Ki-67 labeling index of each component is summarized in Figure 3.

### 2.4. Statistical Analysis

In all immunohistochemical evaluations, the impact of neuroendocrine differentiation on the tumor immune microenvironment in a single patient was analyzed using the Wilcoxon signed-rank test. For pathological cancer-positive lymph nodes (pN) and vessel invasion, the association with each antibody-positive lymphocyte and macrophage was analyzed using the χ^2^ test. The correlation between the number of CD163-positive macrophages infiltrating each region and angiogenesis (indicated by CD34 and VASH-1 expression) was analyzed using the *t*-test. Statistical significance was tentatively set at *p* < 0.05. All statistical analyses were performed using JMP Pro (Ver 16.0.0, SAS Institute, Cary, NC, USA). 

## 3. Results

### 3.1. Clinicopathological Characteristics in the Cases Examined

The clinicopathological characteristics of the cases examined are summarized in Table 1. The sites of GEP-MiNENs were as follows: foregut in 27 (82%), midgut in two (6%), and hindgut in four (12%) cases. The non-NEC components of MiNENs examined in this study included well-differentiated adenocarcinomas in 14 (42%), moderately differentiated adenocarcinomas in 13 (40%), poorly differentiated adenocarcinoma in five (15%), and mucinous carcinoma in one case (3%). Both lymphatic and venous invasions were histologically detected in 20 (60%) cases, and lymph node metastasis was detected in 16 cases (48%).

### 3.2. Difference in TILs between NEC and Non-NEC Components in Intra-Tumoral Areas and Invasive Margins

**Figure 4:**
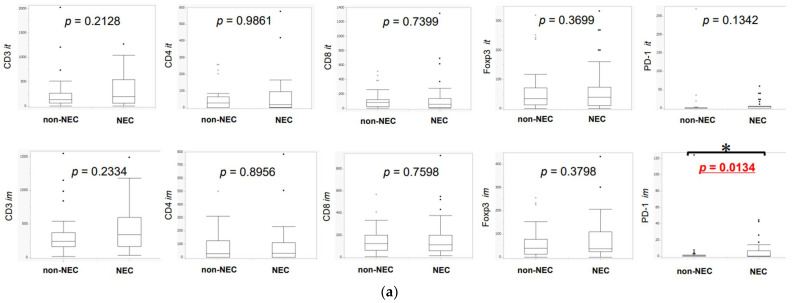

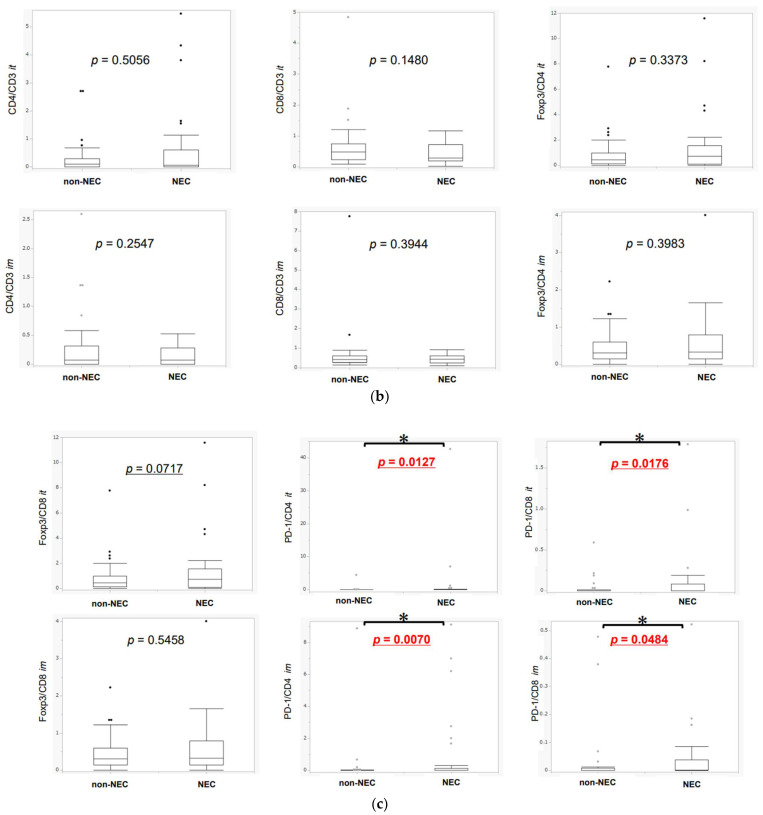
The difference in the abundance of lymphocytes expressing different markers between NEC and non-NEC components in the same case (Wilcoxon signed-rank test). (**a**). PD-1-positive lymphocytes infiltrated into the NEC components more abundantly than the non-NEC components in invasive margins (it: *p* = 0.1342, im: *p* = 0.0134). There were no significant differences in the abundances of CD3- and Foxp3-positive lymphocytes, but more lymphocytes infiltrated into the NEC components than the non-NEC components in both intra-tumoral areas and invasive margins. The points are outliers. * it: intra-tumoral, im: invasive margin; NEC, neuroendocrine; non-NEC, non-neuroendocrine. (**b**). There were no significant differences in the CD4/CD3 and Foxp3/CD4 ratios. However, the CD8/CD3 ratio for infiltrating lymphocytes was lower in the NEC than in the non-NEC component in intra-tumoral areas (CD8/CD3 it: *p* = 0.1430). For infiltrating lymphocytes, it was higher in the NEC component than the non-NEC component in intra-tumoral areas. The points are outliers. * it: intra-tumoral, im: invasive margin. (**c**). The Foxp3/CD8 ratio for infiltrating lymphocytes was higher in the NEC component than the non-NEC component in intra-tumoral areas (Foxp3/CD8 it: *p* = 0.0717, im: *p* = 0.5458). Both PD-1/CD4 and PD-1/CD8 ratios in infiltrating lymphocytes were significantly higher in the NEC component than in the non-NEC component in intra-tumoral areas (PD-1/CD4 it: *p* = 0.0127, im: *p* = 0.0070, PD-1/CD8 it: *p* = 0.0176, im: *p* = 0.0484). The points are outliers. * it: intra-tumoral, im: invasive margin.

The details of TILs and TAMs between NEC and non-NEC components in each intra-tumoral area and invasive margin are summarized in Figure 4a There were no significant differences in the abundance of CD3-, CD4-, CD8-, and Foxp3-positive lymphocytes between the NEC and non-NEC components. The number of PD-1-positive lymphocytes infiltrating into the NEC component was significantly higher than that infiltrating into the non-NEC component, in invasive margins (PD-1 intra: *p* = 0.1342, PD-1 margin: *p* = 0.0134). CD4/CD3, CD8/CD3, Foxp3/CD4, Foxp3/CD8, PD-1/CD4 and PD-1/CD8 ratios were subsequently calculated (Figure 4b,c). PD-1/CD4 and PD-1/CD8 ratios were significantly higher in NEC than in non-NEC components (PD-1/CD4 intra: *p* = 0.0127, margin: *p* = 0.0070; PD-1/CD8 intra: *p* = 0.0176, margin: *p* = 0.0484). CD8/Foxp3 ratio was higher and CD8/CD3 lower in NEC than in non-NEC components, but the tendency did not reach statistical significance (Foxp3/CD8 intra: *p* = 0.0717, CD8/CD3 intra: *p* = 0.1480). There were only seven cases with high PD-L1 status in this study and no significant correlation was detected between the PD-1 and PD-L1 status in non-NEC or NEC components (non-NEC: *p* = 0.5691, NEC: *p* = 0.1762). PD-1-positive lymphocytes were more abundant in NEC than in non-NEC components in 12 (36.4%) and 15 (45.5%) cases in intra-tumoral areas and invasive margins, respectively. CD8-positive lymphocytes were more abundant in NEC than in non-NEC components in 12 (36.4%) and 17 (51.5%) cases, respectively, in those areas. Foxp3-positive lymphocytes were more abundant in NEC than in non-NEC components in 18 (54.5%) and 16 (48.5%) cases and PD-1/CD4 ratio was greater in NEC than in non-NEC components in 12 (36.4%) and 15 (45.5%) cases, respectively. PD-1/CD8 ratio was greater in NEC than in non-NEC components in 13 cases (42.4%), and the Foxp3/CD8 ratio was greater in NEC than in non-NEC components in 19 (57.6%) cases, as summarized in Table 3. Results are summarized in Figures S1–S3.

### 3.3. Difference in TAMs between NEC and Non-NEC Components in Intra-Tumoral Areas and Invasive Margins

**Figure 5:**
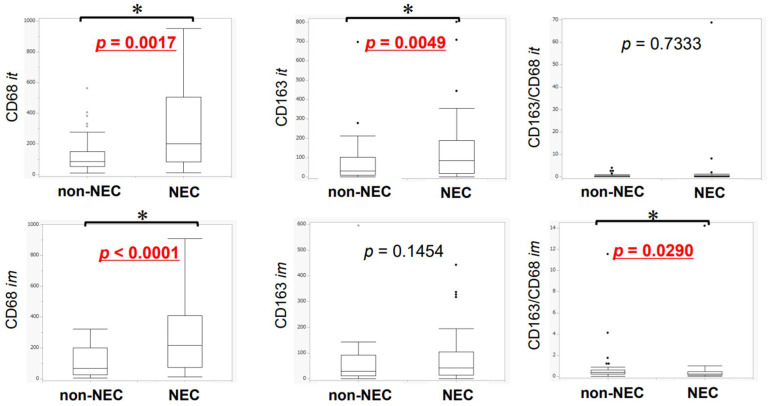
The difference in the abundance of macrophages expressing different markers between the NEC and non-NEC components in the same case (Wilcoxon signed-rank test). CD68- and CD163-positive macrophages infiltrating the NEC components were more abundant than those infiltrating the non-NEC components, the former in both intra-tumoral areas and invasive margins (it: *p* = 0.0017, im: *p* < 0.0001), and the latter only in intra-tumoral areas (it: *p* = 0.0049, im: *p* = 0.1454). CD163/CD68 ratio of infiltrating macrophages was higher in the non-NEC than in the NEC components in the areas of invasive margin (it: *p* = 0.7333, im: *p* = 0.0290). The points are outliers. * it: intra-tumoral, im: invasive margin.

The differences in TAMs between the NEC and non-NEC components in intra-tumoral areas and invasive margins are summarized in Figure 5. In both intra-tumoral areas and invasive margins, CD68-positive macrophages infiltrating into the NEC components were significantly more abundant than those infiltrating into the non-NEC components (CD68 intra: *p* = 0.0017, CD68 margin: *p* < 0.0001). Conversely, CD163-positive macrophages (M2-like macrophages) infiltrating into the NEC component were significantly more abundant than those infiltrating into the non-NEC component in the intra-tumoral areas (CD163 intra: *p* = 0.0049). No significant differences were observed in the invasive margins, but M2-like macrophages tended to harbor greater abundance in the NEC component (*p* = 0.1454). CD163/CD68 ratio was significantly higher in invasive margin in NEC than in non-NEC components (CD163/CD68 margin: *p* = 0.0290) (Figure 5). CD68-positive macrophages were more abundant in NEC than in non-NEC components in 20 (60.6%) and 25 (75.8%) cases, and CD163-positive macrophages were more abundant in NEC than in non-NEC components in 22 (66.7%) and 17 (51.5%) cases examined, in the intra-tumoral areas and invasive margins, respectively. However, the CD163/CD68 ratio was greater in NEC than in non-NEC components in 13 (39.4%) and 10 (30.3%) cases in the intra-tumoral and invasive margins, respectively (Table 3). The results are summarized in Figure S4.

### 3.4. Differences in Neoangiogenesis between NEC and Non-NEC Components

**Figure 6:**
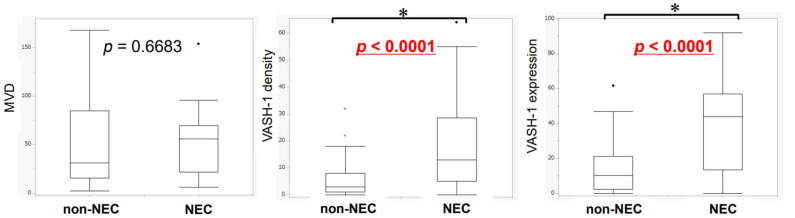
Difference in the number of micro-vessels expressing different markers in NEC and non-NEC components in the same case (Wilcoxon signed-rank test). The number of VASH-1-positive micro-vessels and the ratio of VASH-1 expression were significantly greater in the NEC area (VASH-1 density: *p* < 0.0001, VASH-1 expression: *p* <0.0001). There were no significant differences in the MVD (MVD: *p* = 0.6683). The points are outliers. * it: intra-tumoral, im: invasive margin. MVD, micro-vessel density.

The differences in neoangiogenesis between NEC and non-NEC components are summarized in Figure 6. VASH-1 density and immunoreactivity were significantly higher in the NEC (both *p* < 0.0001) than in the non-NEC components. There were no significant differences in MVD between these two components. MVD, VASH-1 density, and immunoreactivity were greater in NEC than in non-NEC components in 19 (57.6%), 24 (72.7%), and 24 (72.7%) cases, respectively (Table 3). The results are summarized in Figure S5.

### 3.5. Association between Neoangiogenesis and CD163-Positive Macrophage Infiltration in Intra-Tumoral Areas and Invasive Margins in NEC and Non-NEC Components

**Figure 7:**
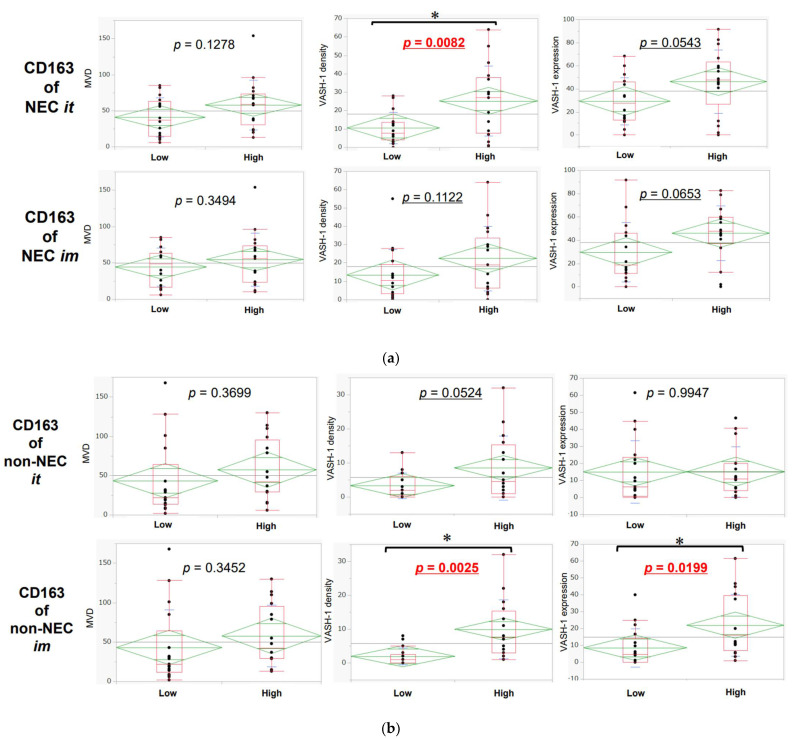
(**a**) Association between the number of CD163-positive macrophages infiltrating intra-tumoral areas and invasive margins in NEC components (*t*-test). The number of CD163-positive macrophages was significantly associated with the VASH-1 density and immunoreactivity and tended to be associated with an increased MVD. The points are outliers. * it: intra-tumoral, im: invasive margin; cutoff: median. (**b**). Association between the number of CD163-positive macrophages infiltrating the intra-tumoral areas and invasive margins in non-NEC components (*t*-test). The high infiltration of CD163-positive macrophages was significantly associated with the VASH-1 density and immunoreactivity, but not with the MVD. The points are outliers. * it: intra-tumoral, im: invasive margin; cut off: median.

In the high CD163 expression group, VASH-1 density and immunoreactivity were greater in the NEC than in the non-NEC components in both intra-tumoral areas and invasive margins, but the difference was not statistically significant (Figure 7a,b).

### 3.6. Association of TILs or TAMs with Lymph Node Metastasis, Lymphatic Invasion, and Venous Invasion

The association of the abundance of lymphocytes infiltrating into the intra-tumoral areas and invasive margins and VASH-1 f to lymph node metastasis and vascular invasion are summarized in Tables S1 and S2. Only CD4 status was significantly higher in the intra-tumoral areas of the NEC than in the non-NEC components (*p* = 0.0229).

## 4. Discussion

This is the first study to evaluate the differences of the tumor microenvironments of neuroendocrine and non-neuroendocrine components in the patients with MiNENs. The results first demonstrated that the status of intra-tumoral infiltration of Foxp3- and CD8-positive lymphocytes was not significantly different between NEC and non-NEC components, but CD8/Foxp3 ratio tended to be higher in NEC components. Konno et al. previously reported that the Foxp3/CD8 ratio was correlated with prognosis of the patients with esophageal squamous cell carcinoma who underwent neoadjuvant chemotherapy (5-fluorouracil+cisplatin) [35]. In addition, Katz et al. also reported that CD8- and Foxp3-positive lymphocytes themselves had no prognostic impact [36]. The status of VASH-1, which represents the neoangiogenesis status, was also reported to suppress TILs and anti-tumor immune responses [37]. In addition, there were significant increments of PD-1/CD4 and PD-1/CD8 ratios both in intra-tumoral and invasive margins. Lim et al. reported that high lymphocytic ratio of PD-1/CD8 resulted in worse prognosis in patients with extrahepatic bile duct carcinoma [38]. Pardoll et al. reported that high ratio of PD-1/CD8 was observed to suppress anti-tumor immune responses [39]. Therefore, increased Foxp3/CD8, PD-1/CD4, and PD-1/CD8 ratios and VASH-1 expression in the tumor tissues were considered to suppress anti-tumor immunity more markedly in NEC than in non-NEC components of MiNENs. These results also suggest the difference in the tumor microenvironment between these two components. The current cohort did not include the cases of MiNENs in the appendix and small intestine, but MiNENs arising in the appendix and small intestine were reported to be associated with poorer clinical outcome compared to those in other sites of the GI tract or pure NECs arising in the appendix or small intestine [13], although the clinical outcomes of MiNENs arising in other sites of the GI tract have been reported to be similar to those of pure NECs arising in the same organ [13]. In addition, the small intestinal epithelium was reported to contain comparable levels of Foxp3-positive lymphocytes as the lymph nodes and to be associated with an immunosuppressive response [40]. The abundance of Foxp3-positive lymphocytes in the appendix was also reported to increase under inflammatory conditions [41]. These findings in the small intestine and appendix could be related to the fact that lymphoid tissue is relatively abundant in both organs, and this could be intrinsically associated with a more pronounced tumor immune suppression; however, further investigations are required for clarification. The therapeutic approach to MiNENs has not necessarily been established, but is generally considered to be governed by the component with a higher grade in the tumor tissues [9]. However, an effective therapeutic approach to one of the two MiNEN components could disrupt the balance of the tumor microenvironment and induce the growth of the other tumor component [10,42]. The results of our present study also indicated the differences between the tumor immune microenvironments of the two components of MiNENs. The therapeutic approaches for several malignancies at this juncture could involve the effective inhibition of in situ tumor immune suppression caused by cancer cells. In this regard, PD-1-positive lymphocytes were more abundant in NEC than in non-NEC components in more than 35% of the MiNENs examined, both in intra-tumoral areas and invasive margins of the tumor (*p* = 0.1342 and *p* = 0.0134, respectively). In addition, Foxp3/CD8 ratio tended to be greater in NEC than in non-NEC components of the MiNENs examined and PD-1/CD4 and PD-1/CD8 ratios were significantly higher in NEC. These findings also indicated that the suppression of local anti-tumor immunity could be more pronounced in NEC than in non-NEC components in MiNENs and the effects of immune checkpoint inhibitor could be less pronounced in NEC components in MiNENs but further investigations are required for clarification.

Both CD68, which serves as pan-macrophage marker, and CD163, which represents M2-like macrophages as well as positive macrophages were significantly more abundant in NEC than in non-NEC components in MiNENs in this study. Sawa and Yu-jie independently reported that humoral factors and the neuroendocrine differentiation of colorectal carcinoma cells induced macrophage differentiation and promoted the production of various chemokines, including VEGF [43,44]. Therefore, the equilibrium of macrophages could also contribute to the local anti-tumor immunity status [45]. In our present study, both pan- and M2-like macrophages were more abundant in the NEC components, possibly owing to the presence of cytokines or growth factors secreted from neuroendocrine granules, as previously reported [43,44]. Both CD68- and CD163-positive macrophages were significantly higher in the areas of both intra-tumoral and invasive margin, while the ratio of CD163/CD68 was significantly lower in invasive margin of NEC component. Therefore, further investigations are warranted to explore the contribution of macrophages to the suppression of anti-tumor immunity in carcinoma cells undergoing neuroendocrine differentiation. In this study, we also examined the neovascularization status, which is an important component of the local tissue microenvironment. The results demonstrated that VASH-1 positivity in the NEC components was greater than that in the non-NEC components of the same MiNEN cases, but was not necessarily associated with lymphovascular invasion and/or lymph node metastasis. However, in cases with high CD163 status, VASH-1 was more pronounced, especially in the NEC component. CD163 is generally considered to enhance angiogenesis [46], whereas VASH-1 is considered to suppress angiogenesis [47]. Therefore, growth factors or other components secreted from neuroendocrine granules could influence the negative feedback of VASH-1, and the tissue turnover could thus be enhanced in angiogenesis in the tumors with neuroendocrine differentiation.

There were some limitations to this study. First, MiNENs are extremely rare tumors, especially when strictly evaluated, and the number of the cases available for examination in this study (33) could be considerably too small to draw definitive conclusions regarding the potential effects of neuroendocrine differentiation on the localized tumor microenvironment. Second, the neuroendocrine components of MiNENs were NECs and not NETs. Currently, NETs and NECs are considered to be distinct tumor types with both morphological and biological differences, including differences in mutation profiles [48,49]. Therefore, it is also important to note that in this study, we evaluated the effects of NECs and not those of general neuroendocrine differentiation.

## 5. Conclusions

This study is the first to demonstrate the differences in the tissue microenvironment between NEC and non-NEC components of MiNENs and provide insights into the effects of neuroendocrine differentiation on tumor microenvironments. The results of this study provide information that may be important for administering anti-tumor immune therapy to the patients with MiNENs.

## Figures and Tables

**Table 1:** Summary of the clinicopathological characteristics of the patients with mixed neuroendocrine non-neuroendocrine neoplasm in this study.

**Number of Patients**	GEP	33
**Age (Years)**	Mean (Range)	72 (56-86)
**Sex (%)**	Male (%)	26 (79)
	Female (%)	7 (21)
**Tumor Location (%)**	Foregut (%)	27 (82)
	Midgut (%)	2 (6)
	Hindgut (%)	4 (12)
**Treatment (%)**	Operation (%)	31 (94)
	ESD (%)	2 (6)
**Histopathological Type (NEC) (%)**	Small cell type (%)	20 (60)
	Large cell type (%)	13 (40)
**Histopathological Type (non-NEC) (%)**	well differentiated (%)	14 (42)
	moderately differentiated (%)	13 (40)
	poorly differentiated (%)	5 (15)
	mucinous (%)	1 (3)
**Lymphatic Invasion (%)**	ly− (%)	13 (40)
	ly+ (%)	20 (60)
**Venous Invasion (%)**	v− (%)	13 (40)
	v+ (%)	20 (60)
**pT (%)**	T1-2 (%)	22 (67)
	T3-4 (%)	11 (33)
**pN (%)**	pN− (%)	17 (52)
	pN+ (%)	16 (48)

Abbreviations: GEP gastroenteropancreatic.

**Table 2:** Summary of the immunohistochemistry protocols used in this study.

Antibody	Vendor	Host	Antigen Retrieval	Dilution	Buffer pH	Reaction Time of Primary Antibody	Secondary Antibody
CD3	DAKO	Rabbit	AC, 121 °C, 5 min	Ready to use	9.0	4 °C, overnight	EnVision FLEX
CD4	Nichirei	Mouse	AC, 121 °C, 5 min	1/80	9.0	4 °C, overnight	Histofine
CD8	DAKO	Mouse	AC, 121 °C, 5 min	1/50	7.0	4 °C, overnight	EnVision FLEX
CD34	Nichirei	Mouse	none	1/200	none	4 °C, overnight	Histofine
CD68	DAKO	Mouse	Trypsin (37 °C, 15 min)	1/200	none	4 °C, overnight	Histofine
CD163	Leica microsystem	Mouse	AC, 121 °C, 5 min	1/600	6.0	4 °C, overnight	Histofine
Foxp3	Abcam	Mouse	AC, 121 °C, 5 min	1/200	6.0	4 °C, overnight	Histofine
PD-1	Abcam	Mouse	AC, 121 °C, 5 min	1/100	6.0	4 °C, overnight	Histofine
PD-L1	Roche, sp263	Rabbit	unknown	RTV	unknown	unknown	VENTANA, Optiview DAB Universal Kit
SYN	DAKO	Mouse	AC, 121 °C, 5 min	1/300	6.0	4 °C, overnight	Histofine
ChgA	Agilent technologies	Rabbit	Microwave (210 W, 15 min)	1/1500	6.0	4 °C, overnight	Histofine
INSM1	Santacruz	Mouse	AC, 121 °C, 5 min	1/200	6.0	4 °C, overnight	Histofine
Ki-67	DAKO	Mouse	PT Link (97 °C, 20 min), Target Retrieval Solution	Ready to use	High pH	20 min, room temperature	EnVision FLEX
VASH-1	Donated	Mouse	AC, 121 °C, 5 min	1/400	8.0	4 °C, overnight	Histofine

Abbreviations: CD cluster of differentiation, AC autoclave, PD-1 programed cell death-1, PD-L1 programmed cell death ligand-1, SYN synaptophysin, ChgA chromogranin A, INSM1 insulinoma-associated 1, VASH-1 vasohibin-1.

**Table 3:** The difference of each antibody positive lymphocytes, macrophage, and molecules for micro-vessels between NEC and non-NEC and the ratio of the cases with more positive ones in NEC than in non-NEC areas. * median ± SD.

Antibodies	NEC-Non-NEC			
	Intra-Tumoral *	NEC > Non-NEC (%)	Invasive Margin *	NEC > Non-NEC (%)
CD3	9.33 ± 359.25	54.5	43.33 ± 332.71	57.6
CD4	−0.67 ± 142.78	42.4	0 ± 108.94	39.4
CD8	−9.67 ± 277.47	36.4	0.67 ± 199.06	51.5
Foxp3	5.00 ± 75.63	54.5	0.33 ± 68.18	48.5
PD-1	0 ± 38.43	36.4	0 ± 23.76	45.5
CD68	66.00 ± 224.67	60.6	58.67 ± 237.92	75.8
CD163	16.67 ± 154.52	66.7	2.33 ± 103.05	51.5
MVD	4.00 ± 44.38	57.6	-	-
VASH-1 density	9.00 ± 16.77	72.7	-	-
VASH-1 expression (%)	15.00 ± 26.71	72.7	-	-
CD4/CD3	−0.0057 ± 1.18	45.5	−0.0017 ± 0.50	33.3
CD8/CD3	−0.077 ± 0.84	39.4	−0.052 ± 1.33	45.5
Foxp3/CD4	0 ± 34.15	45.5	0.074 ± 26.88	51.5
Foxp3/CD8	0.32 ± 2.82	57.6	0.034 ± 0.77	57.6
CD163/CD68	−0.041 ± 11.63	39.4	−0.14 ± 0.86	30.3
PD-1/CD4	0 ± 2.50	36.4	0 ± 7.55	45.5
PD-1/CD8	0 ± 0.27	42.4	0 ± 0.058	42.4

Abbreviations: NEC neuroendocrine carcinoma, CD cluster of differentiation, MVD micro-vessel density, VASH-1 vasohibin-1, PD-1 programed cell death-1.

## Data Availability

The data in this study are available from the corresponding authors. The data were not made publicly available because of ethical restrictions.

## Supplementary Materials

The following are available online at https://www.mdpi.com/article/10.3390/cancers14092152/s1. Figure S1: Graphs connecting the corresponding points for lymphocytes expressing specific markers in the NEC and non-NEC components in each case; Figure S2: Graphs connecting the corresponding points for lymphocytes expressing specific markers in the NEC and non-NEC components in each case; Figure S3: Graphs connecting the corresponding points for lymphocytes expressing specific markers in the NEC and non-NEC components in each case; Figure S4: Graphs connecting the corresponding points for macrophages expressing specific markers in the NEC and non-NEC components in each case; Figure S5: Graphs connecting the corresponding points for microvessels expressing specific markers in the NEC and non-NEC components in each case; Table S1: The association between the number of lymphocytes infiltrating into intra-tumoral areas and invasive margins in each case (NEC–non-NEC area) in relation to pN (+) and vessel invasion (χ^2^ test); Table S2: The association between the number of macrophages infiltrating into the intra-tumoral areas and invasive margins and VASH-1 (NEC–non-NEC area) in relation to pN (+) and vessel invasion (χ^2^ test).
