# Supplementary material for: Tumor Microenvironment in Mixed Neuroendocrine Non-Neuroendocrine Neoplasms: Interaction between Tumors and Immune Cells, and Potential Effects of Neuroendocrine Differentiation on the Tumor Microenvironment
Source: Cancers (Basel). 2022 Apr 26;14(9):2152. doi: 10.3390/cancers14092152 (PMC9100554; doi:10.3390/cancers14092152)
Supplement: Supplementary file 1 [file cancers-14-02152-s001.zip › cancers-1624141-supplementary.pdf]

Supplementary Materials: Tumor Microenvironment in Mixed Neuroendocrine Non-Neuroendocrine Neoplasms: Interaction between Tumors and Immune Cells, and Potential Effects of Neuroendocrine Differentiation on the Tumor Microenvironment

Junichi Tsunokake, Fumiyoshi Fujishima, Hirofumi Watanabe, Ikuro Sato, Koh Miura, Kazuhiro Sakamoto [5], Hiroyoshi Suzuki, Takashi Sawai, Yuko Itakura, Tatsuya Hoshi, Atsushi Kunimitsu, Takuro Yamauchi, Ryujiro Akaishi, Yohei Ozawa, Toshiaki Fukutomi, Hiroshi Okamoto, Chiaki Sato, Yusuke Taniyama, Takashi Kamei and Hironobu Sasano

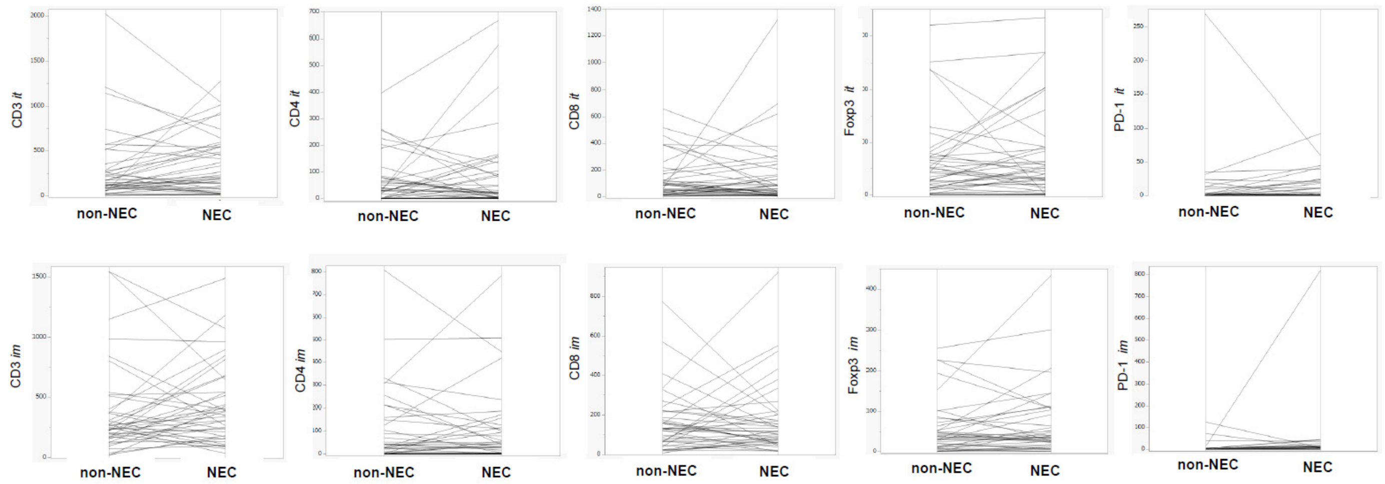

Figure S1. Graphs connecting the corresponding points for lymphocytes expressing specific markers in the NEC and non-NEC components in each case. * it: intra-tumoral, im: invasive margin. NEC, neuroendocrine; non-NEC, non- neuroendocrine.

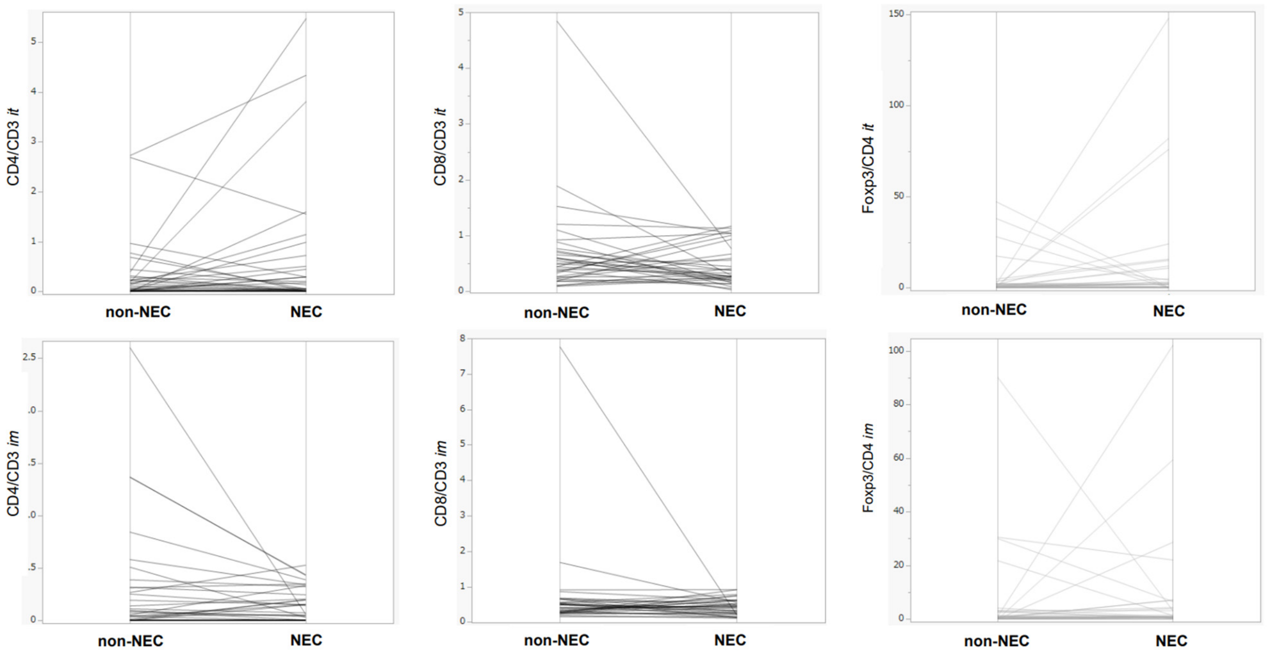

Figure S2. Graphs connecting the corresponding points for lymphocytes expressing specific markers in the NEC and non-NEC components in each case. * it: intra-tumoral, im: invasive margin.

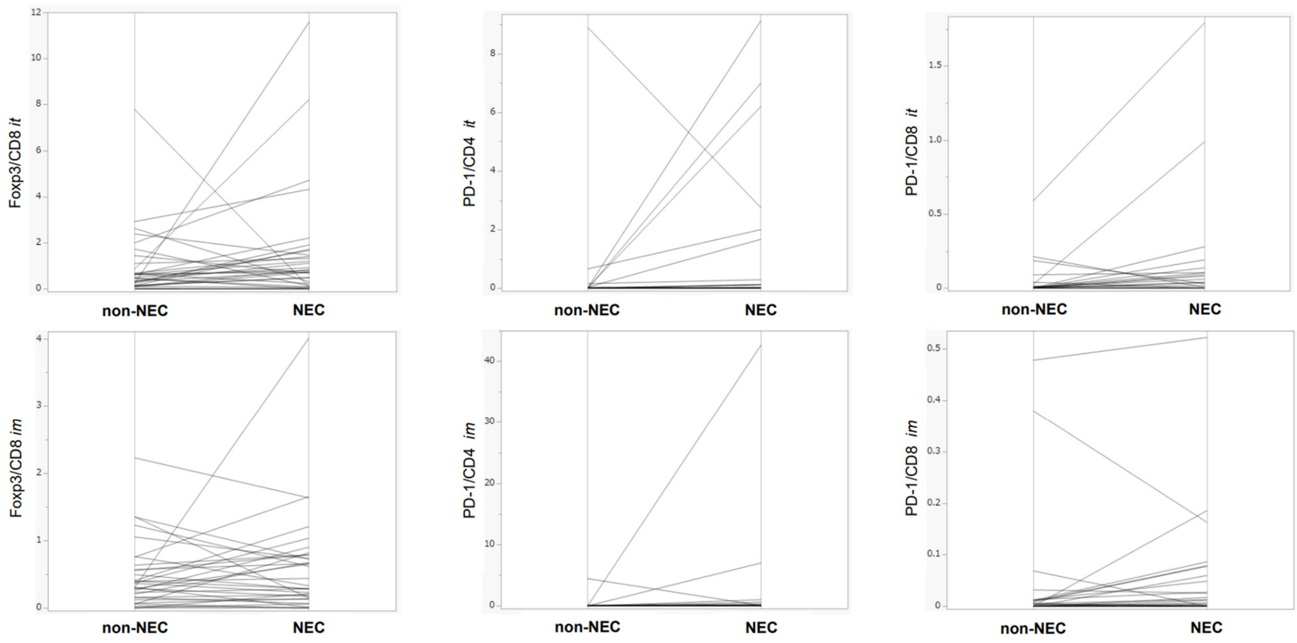

Figure S3. Graphs connecting the corresponding points for lymphocytes expressing specific markers in the NEC and non-NEC components in each case. * it: intra-tumoral, im: invasive margin.

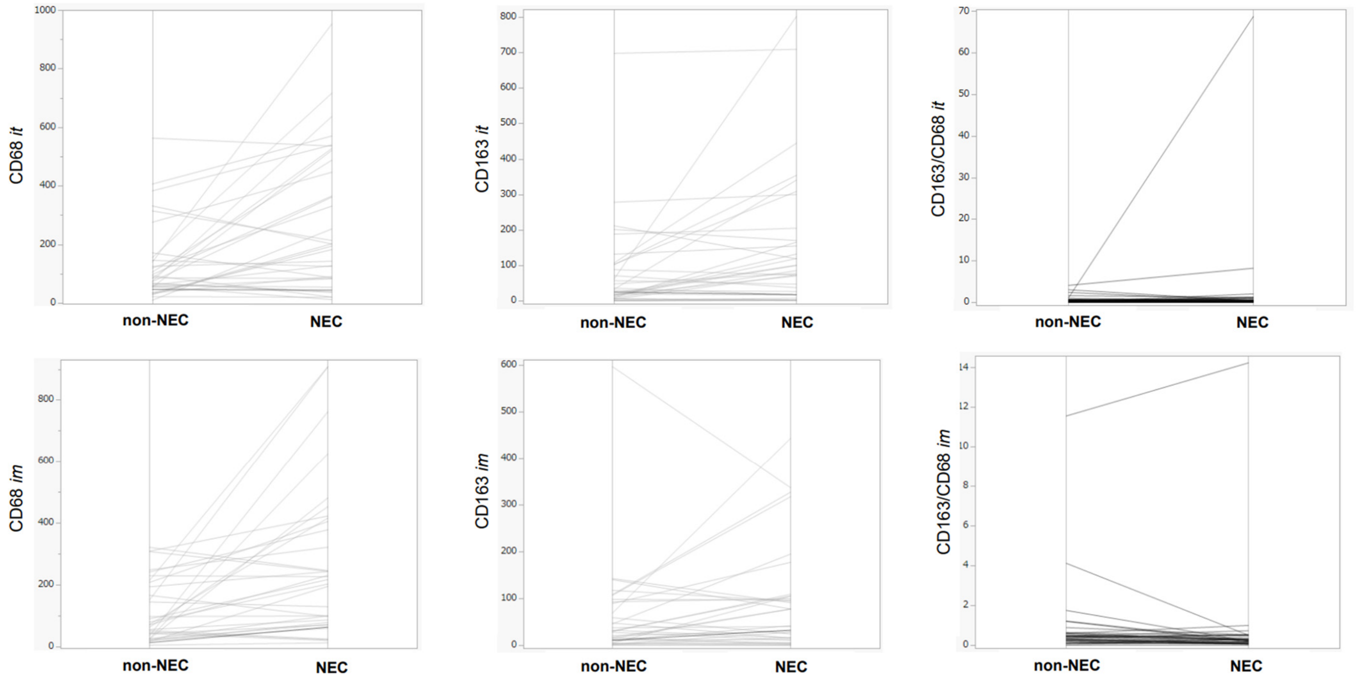

Figure S4. Graphs connecting the corresponding points for macrophages expressing specific markers in the NEC and non-NEC components in each case. * it: intra-tumoral, im: invasive margin.

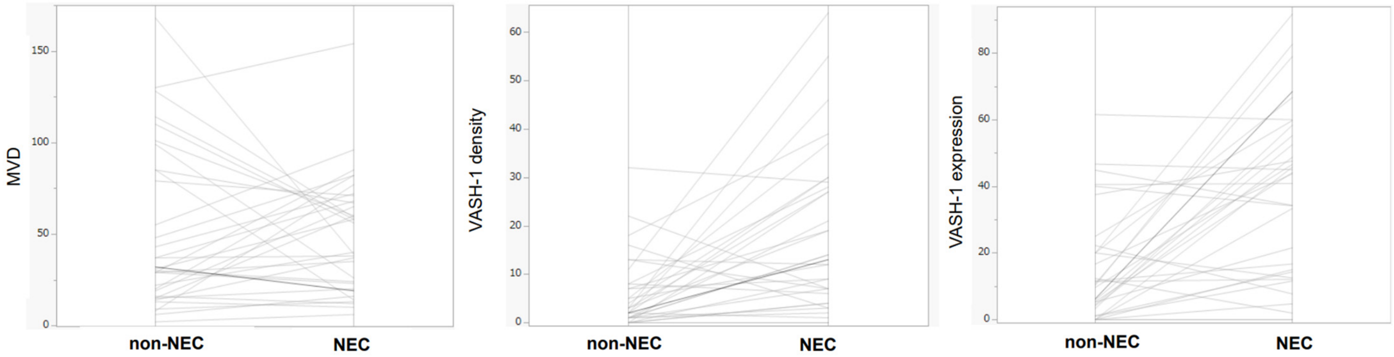

Figure S5. Graphs connecting the corresponding points for microvessels expressing specific markers in the NEC and non-NEC components in each case.

Table S1. The association between the number of lymphocytes infiltrating intra-tumoral areas and invasive margins in each case (NEC–non-NEC area) in relation to pN (+) and vessel invasion (χ^2 test). Only CD4 expression in the intra-tumoral areas and venous invasion in NECs were significantly more abundant than those in non-NECs (p = 0.0229). NEC, neuroendocrine; non-NEC, non- neuroendocrine; pN, pathological cancer-positive lymph nodes.

Antibodies	pN						Ly						V					
	intra-tumoral			invasive margin			intra-tumoral			invasive margin			intra-tumoral			invasive margin		
	pN(+)	pN(-)	*p*	pN(+)	pN(-)	*p*	Ly(+)	Ly(-)	*p*	Ly(+)	Ly(-)	*p*	V(+)	V(-)	*p*	V(+)	V(-)	*p*
CD3																		
high	9	8	0.5975	9	8	0.5975	11	6	0.6193	10	7	0.829	12	5	0.2264	11	6	0.6193
low	7	9		7	9		9	7		10	6		8	8		9	7	
CD4																		
high	6	11	0.1181	8	5	0.2248	11	6	0.6193	8	5	0.9295	9	8	0.353	11	2	0.0229
low	10	6		8	12		9	7		12	8		11	5		9	11	
CD8																		
high	6	11	0.5975	7	9	0.5975	9	8	0.358	9	7	0.6193	10	7	0.829	10	6	0.829
low	10	6		9	8		11	5		11	6		10	6		10	7	
Foxp3																		
high	7	9	0.3921	7	10	0.3865	10	6	0.829	9	8	0.353	10	6	0.829	11	6	0.6193
low	9	8		9	7		10	7		11	5		10	7		9	7	
PD-1																		
high	7	5	0.3921	8	7	0.6109	7	5	0.8399	9	6	0.9481	11	6	0.6193	8	4	0.5902
low	9	12		8	10		13	8		11	7		9	7		12	9	

Table S2. The association between the number of macrophages infiltrating the intra-tumoral areas and invasive margins and VASH-1 expression (NEC–non-NEC area) in relation to pN (+) and vessel invasion (χ^2 test). There were no significant differences between the components. VASH-1, vasohibin 1.

Antibodies	pN						Ly						V					
	intra-tumoral			invasive margin			intra-tumoral			invasive margin			intra-tumoral			invasive margin		
	pN(+)	pN(-)	*p*	pN(+)	pN(-)	*p*	Ly(+)	Ly(-)	*p*	Ly(+)	Ly(-)	*p*	V(+)	V(-)	*p*	V(+)	V(-)	*p*
CD68																		
high	8	8	0.8658	8	8	0.8658	8	8	0.2264	11	5	0.353	8	8	0.2264	10	6	0.829
low	8	9		8	9		12	5		9	8		12	5		10	7	

CD163																		
high	8	8	0.865	9	8	0.597	10	6	0.829	12	8	1	8	8	0.2264	10	7	0.829
low	8	9	8	7	9	5	10	7		12	8		12	5		10	6	
VASH-1 density																		
high	—	—	—	—	—	—	9	7	0.6193	—	—	—	8	8	0.2264	—	—	—
low	—	—		—	—		11	6		—	—		12	5		—	—	
VASH-1 expression																		
high	—	—	—	—	—	—	8	8	0.2264	—	—	—	7	9	0.0545	—	—	—
low	—	—		—	—		12	5		—	—		13	4		—	—	
